# Selection of a Novel DNA Aptamer Specific for 5-Hydroxymethylfurfural Using Capture-SELEX

**DOI:** 10.3390/bios13050564

**Published:** 2023-05-22

**Authors:** Xixia Liu, Yingyu Hou, Yanlin Qin, Jiaxin Cheng, Jianjun Hou, Qin Wu, Zhenmin Liu

**Affiliations:** 1 State Key Laboratory of Dairy Biotechnology, Shanghai Engineering Research Center of Dairy Biotechnology, Dairy Research Institute, Bright Dairy & Food Co., Ltd., Shanghai 200436, China; liuxixia@hbnu.edu.cn; 2 Hubei Key Laboratory of Edible Wild Plants Conservation and Utilization, Hubei Normal University, Huangshi 435002, China

**Keywords:** 5-hydroxymethylfurfural, systematic evolution of ligands by exponential enrichment, high-throughput sequencing technology, aptamers, quenching biosensor

## Abstract

A capture systematic evolution of ligands by exponential enrichment (Capture-SELEX) was described to discover novel aptamers specific for 5-hydroxymethylfurfural (5-HMF), and a biosensor based on molecular beacon was constructed to detect 5-HMF. The ssDNA library was immobilized to streptavidin (SA) resin to select the specific aptamer. The selection progress was monitored using real-time quantitative PCR (Q-PCR), and the enriched library was sequenced by high-throughput sequencing (HTS). Candidate and mutant aptamers were selected and identified by Isothermal Titration Calorimetry (ITC). The FAM-aptamer and BHQ1-cDNA were designed as the quenching biosensor to detect 5-HMF in milk matrix. After the 18th round selection, the Ct value decreased from 9.09 to 8.79, indicating that the library was enriched. The HTS results indicated that the total sequence numbers for 9th, 13th, 16th, and 18th were 417054, 407987, 307666, and 259867, but the number of sequences for the top 300 sequences was gradually increased from 9th to 18th, and the ClustalX2 analysis showed that there were four families with high homology rate. ITC results indicated that the *K*_d_ values of H1 and its mutants H1-8, H1-12, H1-14, and H1-21 were 2.5 μM, 1.8 μM, 1.2 μM, 6.5 μM, and 4.7 μM. The linear range of the quenching biosensor was from 0 μM to 75 μM, and it had a similar linear range in the 0.1% milk matrix. This is the first report to select a novel aptamer specific for 5-HMF and develop quenching biosensor for the rapid detection of 5-HMF in milk matrix.

## 1. Introduction

5-hydroxymethylfurfural (5-HMF) and furfural (FF) are small molecules produced by the Maillard reaction (MR) and caramelization of many sugary foods during processing [1,2,3]. The content of 5-HMF in food increased with the extension of storage time, which could be used as an indicator to evaluate the degree of hot processing and storage [4]. However, our previous study found that the content of FF decreased with the extension of storage time [5]. The scientific panel on food additives, flavors, processing aids, and food contact materials of the European commission for food safety considers that the daily intake limit of 5-HMF is 1.6 mg/person [6]. The concentration of 5-HMF in most milk powder samples was about 10 times higher than FF, and the range of 5-HMF was 0.22 to 1.70 mg/100 g protein. The concentration of 5-HMF was even higher in charcoal yogurt [7,8]. Therefore, the presence of 5-HMF in dairy products may be more important for us to pay more attention to the impact of dietary health.

Studies showed that 5-HMF had pharmacological activities such as antioxidation [9] and neuroprotective effect [10] at low concentration levels. However, at high concentrations, it could also cause oxidative stress [5] and had nephrotoxicity [11] and potential genotoxicity [12,13]. People’s life is often inseparable from milk powder and other milk products, especially related to the growth of infants and teenagers. If its content in milk powder cannot be effectively monitored, 5-HMF will become a food contaminant of great concern to scientists. Therefore, it is necessary to develop a simple, rapid, and sensitive method to determine the content of 5-HMF in milk powder.

Researchers have established a variety of methods to detect the content of 5-HMF in foods. For example, ultra-high-performance liquid chromatography [14], gas chromatography [15], and enzyme-linked immunosorbent assay [16] have been reported for 5-HMF detection. These traditional strategies could provide accurate and reproducible results, but they always need expensive instruments or reagents and highly qualified personnel. Moreover, the sample pretreatment method was complex and time-consuming and had low detection efficiency. So, a few sensors have been constructed for 5-HMF detection, such as the colorimetric chemosensor [17], electrochemical sensor [18], and fluorescent techniques sensor [19,20]. However, the electrode modification process of the electrochemical sensor is complicated and unstable. The colorimetric sensor is easy to operate but not very sensitive. Fluorescence biosensors are attractive because they enable quick and easy field detection and have high affinity and specificity.

Aptamer is a single-strand DNA (ssDNA) with selective molecular binding properties and particularly useful for recognizing small molecules [21,22]. It was isolated using a combinatorial method called systematic evolution of ligands by exponential enrichment (SELEX) and has the advantages of low cost, high stability, and easy modification [23,24]. In recent years, aptamers recognizing small molecules are gradually screened and widely used in the construction of fluorescent quenching biosensors. The fluorescence quenching biosensor is a non-radiative energy transfer process. Its efficiency mainly depends on the distance between the fluorescence molecule and the quencher molecule [25]. It has been used to construct biosensors to detect many small molecules with the advantages including simple, fast response and high sensitivity. Huang and Liu [26] designed a structure-switching fluorescence quenching sensor based on aptamer to recognize caffeine (the detection limit was 1.2 μM). It has been applied well in the actual sample detection. In our previous study, fluorescence quenching biosensors based on dopamine-recognizing aptamers were also sensitive [27]. To date, no aptamers specific for 5-HMF were reported.

In this study, we firstly conducted the capture-SELEX for selection of the aptamer specific for 5-HMF. Then, the aptamer with high affinity was discovered by high-throughput sequencing (HTS), sequence alignment, secondary structure prediction, and isothermal titration calorimetry (ITC) identification. Finally, we used this aptamer to construct a quenching biosensor for the detection of 5-HMF in milk samples.

## 2. Materials and Methods

### 2.1. Chemicals and Reagents

The 5-HMF and FF standard were purchased from TMRM (Beijing, China). Taq polymerase and dNTPs were purchased from Takara. Streptavidin agarose resin was purchased from smart-life sciences (Changzhou, China). Evagreen was purchased from Shanghai Open Biotechnology Ltd. (Shanghai, China). Amicon Ultra-0.5 centrifugal filter units (3K and 10K) were purchased from Merk. Micro bio-spin chromatography columns were from Bio-Rad. The aptamer library was synthesized by Sangon Biotech Ltd. (Shanghai, China). The primers (F-Lib30, R-Lib30, FAM-F(Lib30), Biotin-R(Lib30), (lib30)CS-Biotin), aptamers, labeled aptamers, and DNA were synthesized by Nanjing Genscript Biotechnology Ltd. (Nanjing, China). These sequences are listed in Table S1. High-throughput sequencing was performed by Anhui Aptamer Biotechnology Ltd. (Hefei, China). The detailed aptamer and mutants for identification are shown in Table 1. All other analytical pure chemical reagents were purchased from Sinopharm Group Chemical Reagents Ltd. (Shanghai, China).

### 2.2. Selection of Aptamers Specific for 5-HMF Based on SELEX

**Figure 1:**
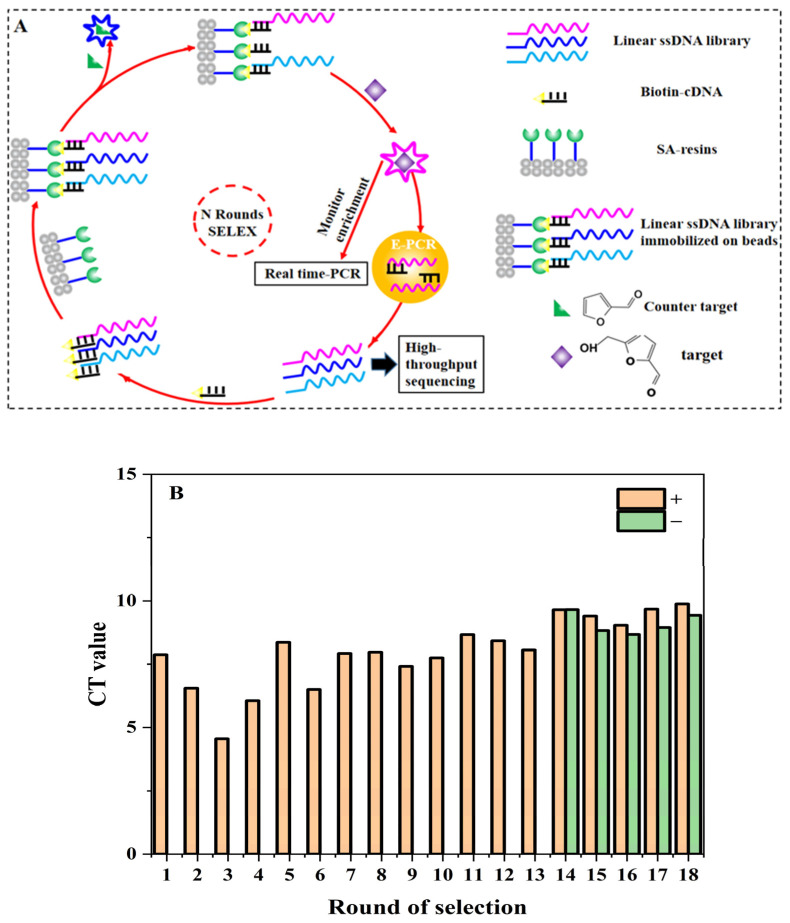
SELEX for 5-HMF aptamers: (**A**) SELEX procedure. (**B**) Selection progress tracking via Q-PCR.

The procedure of SELEX for aptamers against 5-HMF is shown in Figure 1A. The method was based on the research of Huang and Liu [26] with a few modifications. The synthetic ssDNA library (N30Library) was dissolved in SELEX buffer (500 mM NaCl, 10 mM MgCl_2_, and 50 mM HEPES at pH 7.6). The (lib30)CS-Biotin was mixed with the library at a ratio of 3:1 and slowly denatured and renatured (95 °C for 10 min, 68 °C for 1 min, 25 °C for 1 min). The annealed DNA was stored in the ice bath. At the same time, 250 μL streptavidin agarose resin was loaded into the microchromatography column and washed with 500 μL SELEX buffer for 5 times. The annealed DNA was cycled through the column 6 times (3 min each time). In order to remove unbound or loosely bound libraries, the column was washed 12 times with 500 μL SELEX buffer. Then, 740 μL of 5-HMF solution was added, and the eluted DNA was collected by gravity flow. The real-time quantitative (Q-PCR) was used to monitor the selection progress with F-lib30 and R-lib30 as primers. The elution was added to a 3 kDa amicon ultra-0.5 tube in batches for concentration and washed with ultrapure water. The final volume of purified DNA was adjusted to 110–120 μL. Then, all the purified DNA was used as template to prepare the secondary library. We added FF as counter target from the 14th to 18th round. The detailed screening conditions are shown in Table S2.

### 2.3. Establishment of a Q-PCR Method for the Monitoring Selection Process

A standard curve was prepared as follows: the initial ssDNA library was diluted to the concentration (10,000 pM–1 pM) as the Q-PCR templates. Then, 2 μL of template was mixed with 18 μL Q-PCR mix (5 μL F-Lib30 (100 μM), 5 μL R-Lib30 (100 μM), 80 μL dNTP mix (2.5 mM), 100 μL 10× PCR buffer, 25 U Taq DNA polymerase, 50 μL Evagreen, 755 μL sterilization ultrapure water, total volume of 1 mL), and the following Q-PCR program was used: 94 °C 2 min, 94 °C 15 s, 60 °C 30 s, 72 °C 45 s, 25 cycles. The enriched libraries from each round were monitored using Q-PCR according to the above method, and 2 μL of elution library was used as the template.

### 2.4. Preparation of Secondary Libraries

After each round of selection, all of the purified DNA was added into the 2 mL PCR mix (10 μL FAM-F(Lib30) with the concentration of 100 μM, 10 μL Biotin-R(Lib30) with the concentration of 100 μM, 160 μL dNTP mix with the concentration of 2.5 mM, 200 μL 10× PCR buffer, 50 U Taq DNA polymerase, 1610 μL sterilization ultrapure water, total volume of 2 mL) for PCR amplification. The PCR parameters were as follows: 94 °C 4 min, 94 °C 1 min, 60 °C 1 min, 72 °C 1 min, 25 cycles, 72 °C 5 min, 25 °C 1 min. Then, the PCR products were added into a 10 kDa amicon ultra-0.5 tube and centrifuged at 12,000 r/min for 15 min with 4 times. The concentrated mixture was washed using the separation buffer (250 mM NaCl, 50 mM HEPES, pH 7.5). Then, 250 μL of streptavidin agarose resin was washed 5 times with separation buffer. The concentrated PCR product was loaded onto the column and washed with 500 μL separation buffer 10 times. Then, 660 μL of 0.2 M NaOH was added and incubated for 15 min, and the ssDNA library was collected. The library was neutralized with HCl and then added to a 3 kDa amicon ultra-0.5 tube for desalting with 300 μL water. Finally, the SELEX buffer was added to exchange water, and the secondary library was collected with volume of 60 μL. The concentration of ssDNA library was quantified using a NanoDrop spectrophotometer, and the next round of selection was conducted.

### 2.5. High-Throughput Sequencing and Sequence Analysis of the Enrichment Library

The 9th, 13th, 16th, and 18th round libraries and SELEX buffer for control were used to prepare sequencing template with five different primers, which contained a unique index sequence. The prepared sequencing libraries were sent to Anhui Aptamer Biotechnology Co., Ltd. for HTS with an Illumina HTS platform (HiSeq). Homologous alignment was carried out for the top 300 sequences of high-frequency using ClustalX2 software v.2.0. Then, the sequences from high homologous families were selected for secondary structure prediction with DNAMAN software v.6.0. Five aptamers from four families were used to identify affinity with ITC analysis.

### 2.6. Isothermal Titration Calorimetry Analysis

Aptamers and 5-HMF were dissolved in SELEX buffer, and all solutions were degassed for 10 min before each measurement to avoid bubble formation. 10 µM of aptamers were loaded into ITC cell chambers (0.35 mL), and 5-HMF (0.33 mM, 50 μL) was loaded to syringe. Except for the initial injection of 1 μL, 2 μL of the target was titrated into the cell each time over 30 s duration for a total of 25 injections at 25 °C. The spacing was set for 180 s between each injection. Thermodynamic values were obtained by fitting the titration curves to a one-site binding model using the origin software. Then, the mutant aptamers were designed according to the aptamer with the lowest *K*_d_ value.

### 2.7. The Quenching Biosensor Assay

We labeled 6-FAM at the 5′ end of H1-8 (named FAM-H1-8) and BHQ1 at 3′ end of complemented short DNA (named BHQ1-cDNA) to construct quenching biosensor. The FAM-H1-8 (1 μM) was incubated with a serious ratio (1:1, 1:2, 1:3) of BHQ1-cDNA (1 μM) to study the optimal ratio by scanning the fluorescence emission spectra from 500 nm to 650 nm with excitation of 475 nm using an i3X instrument (Molecular Device, CA, USA). Then, 5-HMF titration experiments were performed under optimal conditions. Then, 1 μL FAM-H1-8 (1 μM) and 2 μL BHQ1-cDNA (1 μM) were added to 47 μL SELEX buffer at 95 °C for 5 min. Then, 50 μL a series of 5-HMF concentrations (0–2000 μM) added to SELEX buffer and reacted for 30 min at room temperature. The fluorescence emission spectra from 500 nm to 650 nm were scanned with excitation of 475 nm. Finally, to evaluate the specificity of quenching biosensor, FAM-H1-8 and BHQ1-cDNA were incubated with SELEX buffer at 95 °C for 5 min, and the mixture was reacted with 5-HMF and its analogue FF from 0 μM to 100 μM at room temperature for 30 min. The fluorescence intensity at 520 nm was recorded with excitation of 475 nm.

The milk powder was dissolved in SELEX buffer with concentration of 0.1%, 1%, and 10%, and the samples were centrifuged for 15 min at 12,000 r/min with 4 °C. The supernatant was used as the sample matrix, and then, different concentrations of 5-HMF (0–200 μM) were added to the matrix. The comparisons between the buffer and different concentrations of matrix proceeded using quenching biosensor.

We also designed another molecular beacon to verify the opening or closing status at the 3′ and 5′ end of aptamer. We labeled 6-FAM at the 5′ end and BHQ1 at 3′ end of H1-8 (named F-H1-8-Q). The FAM-H1-8-BHQ1 was incubated with 5-HMF for 30 min, and then the fluorescence emission spectra from 500 nm to 650 nm were scanned with excitation of 475 nm.

## 3. Results

### 3.1. SELEX for 5-HMF Aptamers

The SELEX procedure is shown in Figure 1A. The structures of 5-HMF and FF are also shown in Figure 1A. The quantitative standard curve of Q-PCR is shown in Figure S1, and the enrichment result is shown in Figure 1B. Our main goal was to obtain aptamers specific for 5-HMF through a total of 18 rounds. The counter selections against FF were introduced from the 14th round to improve specificity for 5-HMF. The Q-PCR was used to monitor the selection process for each round according to the Ct values and amplification curve for qualitative and quantitative analyses. At the end of the 13th round, the average Ct value was decreased from 8.42 to 8.05. After the addition of counter selection, the Ct values increased, because some non-specific aptamers were screened and removed. After counter selection, the Ct value of the 16th round was also significantly reduced, which indicated that the enriched aptamer library was specific for 5-HMF (see Figure 1B). Later, we continued to enhance the concentration of FF to screen high affinity aptamers, and the Ct values for the 17th and 18th were stable, indicating that the library was enriched.

### 3.2. High-Throughput Sequencing and Sequences Analysis of the Enriched Library

**Figure 2:**
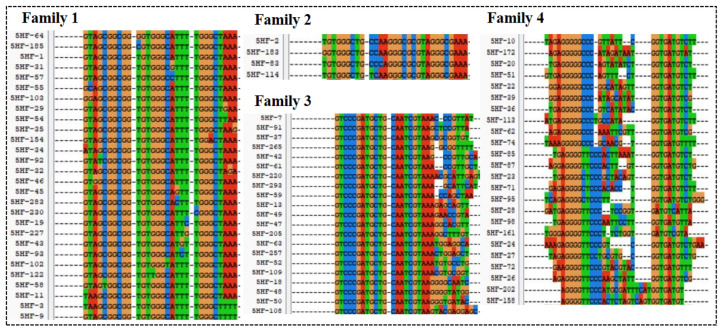
Sequences alignment.

**Figure 3:**
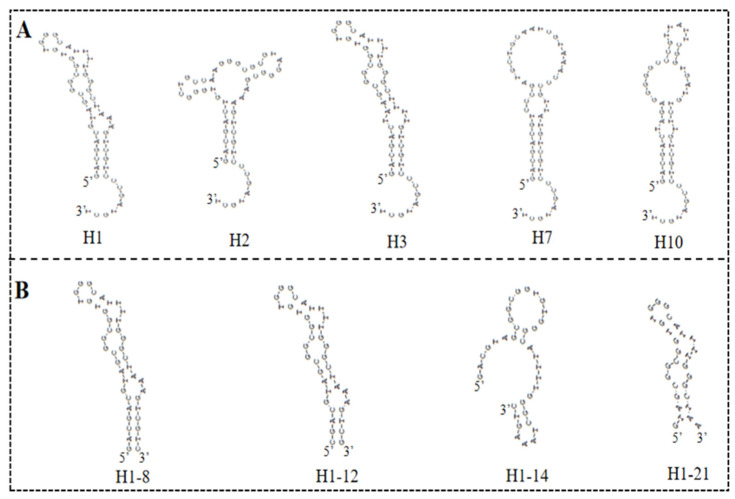
Secondary structures: (**A**) secondary structures of the five selected aptamers. (**B**) Secondary structures of the truncated H1 aptamer.

The high-throughput sequencing technology was used to sequence the enriched library of the 9th, 13th, 16th, and 18th rounds. The top 300 sequences of the high-frequency sequences were selected to analyze their homology rate for the 30 bp random sequences using ClustalX2 software. The results are shown in Figure 2, and the high homologous sequences belong to four families. Then, we selected five aptamers (5HF-1 as H1, 5HF-2 as H2, 5HF-3 as H3, 5HF-7 as H7, and 5HF-10 as H10) to cut their partially fixed area (12 nt and 10 nt were removed from the 5′ and 3′ constant regions of the aptamers), and the detailed sequences information is shown in Table 1. The secondary structures of the tailored aptamers were predicted using DNAMAN software. The results (Figure 3A) showed that these aptamers contained one to three ring structures, which were selected to identify binding activity.

### 3.3. The Affinity Analysis of Aptamers and Its Mutants Using ITC

**Figure 4:**
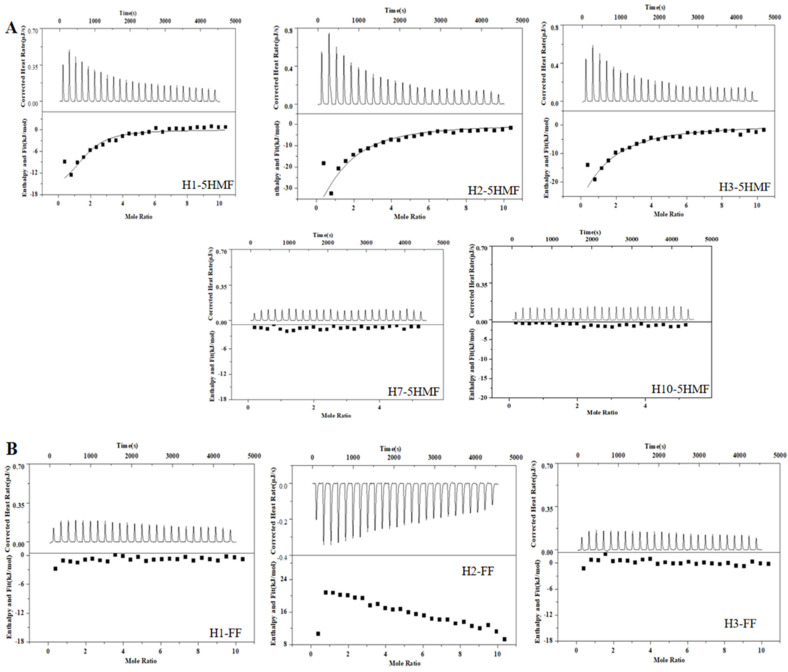
ITC analysis affinity between aptamers and 5-HMF, FF. (**A**) ITC analysis affinity between aptamers and 5-HMF. (**B**) ITC analysis affinity between aptamers and FF.

ITC is a widely used technology for characterization of binding activity between aptamer and small molecule. This technology is label-free, and we did not modify the aptamer candidates. So, the affinity of the selected five aptamers was determined using ITC (Figure 4A), and the corresponding *K*_d_ values were 2.46 ± 1.90 μM for H1 aptamer, 8.76 ± 2.50 μM for H2 aptamer, and 12.40 ± 2.70 μM for H3 aptamer, respectively. The H1 aptamer had the highest affinity to 5-HMF, and its affinity was 2-fold to the H2 aptamer and 3-fold to the H3 aptamer. However, we could not monitor the affinity between 5-HMF and H7 and H10 aptamers, indicating that the H7 and H10 aptamers had no binding activity to 5-HMF. In order to verify the specificity, the FF was titrated to the H1, H2, and H3 aptamers. The results are shown in Figure 4B, and we could not discover the heat change similar to the specific binding, indicating that the H1, H2, and H3 aptamers had no cross-reaction with FF, and H1, H2, H3 aptamers had high specificity for 5-HMF.

**Figure 5:**
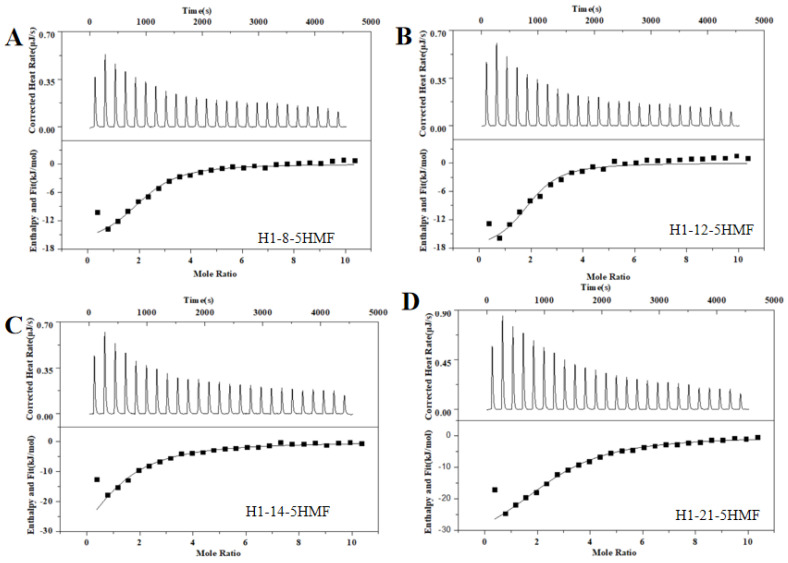
ITC analysis affinity between H1 mutants and 5-HMF. (**A**) H1-8 aptamer. (**B**) H1-12 aptamer. (**C**) H1-14 aptamer. (**D**) H1-21 aptamer.

There is an interesting phenomenon that the secondary structure difference between H1 and H3 aptamer was the sequence for only one ring region, and the sequences were GT…AAA for H1 aptamer and TA…TTT for H3 aptamer. However, the affinity of H1 aptamer was 3-fold to the H3 aptamer. We proposed that the GT…AAA ring is one of the critical domains for binding between aptamer and 5-HMF. In order to verify this idea, we designed the mutants H1-8, H1-12, H1-14, and H1-21, and their secondary structures are shown in Figure 3B. The ITC results (Figure 5) indicated that the *K*_d_ values were 1.79 ± 0.60 μM, 1.22 ± 0.90 μM, 6.50 ± 1.80 μM, and 4.7 ± 0.6 μM for H1-8, H1-12, H1-14, and H1-21, respectively. Compared with the H1 aptamer, the *K*_d_ decreased for H1-8 and H1-12, because we cut the fixed area, and the shortened aptamers of H1-8 and H1-12 had higher affinity. When we continued to cut the H1 aptamer to the H1-14 and H1-21 aptamers, the *K*_d_ enhanced for H1-14 and H1-21, indicating that the affinity decreased. Compared with H1 aptamer, one of the ring regions of the secondary structures for H1-14 and H1-21 aptamers was broken. These results indicated that the GT…AAA ring region is critical for binding between H1 and 5-HMF.

### 3.4. The Quenching Biosensor Assay

**Figure 6:**
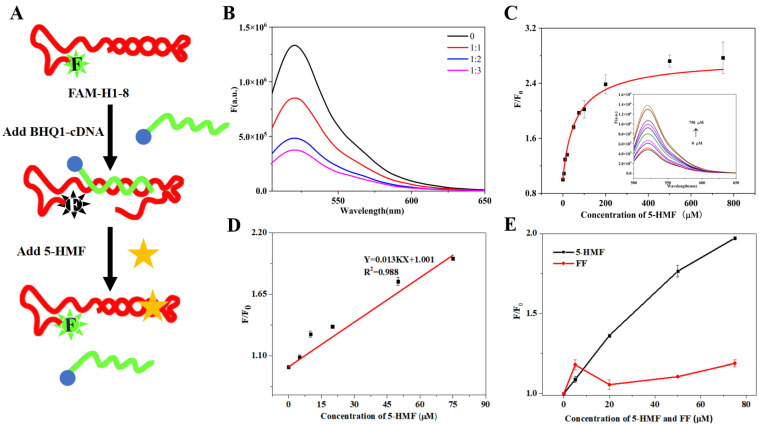
The fluorescence quenching biosensor. (**A**) Principles of the quenching biosensor. (**B**) Optimization of the ratio between FAM-H1-8 and BHQ1-cDNA. (**C**) 5-HMF titrated biosensor. (**D**) The linear curve of 5-HMF. (**E**) The specificity of H1-8.

The principle of the quenching biosensor is shown in Figure 6A. When 5-HMF was absent, the FAM-H1-8 bound with BHQ1-cDNA, and then FAM closed to BHQ1, which can result in the decreasing of fluorescence signal. When 5-HMF was present, the FAM-H1-8 bound with 5-HMF, and the FAM was far from BHQ1, which can result in the recovery of fluorescence signal.

We firstly optimized the ratio between FAM-H1-8 and BHQ1-cDNA. The results are shown in Figure 6B. Compared with the control group (without BHQ1-cDNA), the fluorescence intensity of other groups (with BHQ1-cDNA) was sharply decreased, indicating that BHQ1 quenched the fluorescence of 6-FAM. When the ratios between FAM-H1-8 and BHQ1-cDNA were 1:2 and 1:3, the change of fluorescence intensity was not obvious. In order to save on cost, we selected the ratio of 1:2 between FAM-H1-8 and BHQ1-cDNA for the subsequent experiments.

We performed 5-HMF titration experiments using FAM-H1-8 and BHQ1-cDNA as the quenching biosensor. Under the optimal conditions, the fluorescence intensity increased with increasing 5-HMF concentration (Figure 6C), indicating that H1-8 can bind with 5-HMF. Then, we calculated the *K*_d_ value of 64 μM, which was higher than the *K*_d_ value of ITC. It may be due to the difference of sensitivity between the quenching biosensor and ITC. Then, we further studied the relationship between F/F_0_ and 5-HMF, and the results are shown in Figure 6D. The linear range was from 0 μM to 75 μM, and the limit of detection (LOD) was 11.80 μM which was lower than the residue limit threshold in food (317 μM). Therefore, our biosensor is suitable for 5-HMF detection in food. The comparison of our method for detecting 5-HMF with previously reported methods is shown in Table 2. The obvious advantage of our study is the short detection time.

To evaluate the specificity of the quenching biosensor, 5-HMF and its analog FF were used to titrate the biosensor, and the results are shown in Figure 6E. Compared with the 5-HMF group, the fluorescence signal of the FF group did not display a linear relationship to FF concentration, which indicated that the quenching biosensor has no cross-reactivity with FF. This result verified the specificity of this biosensor.

### 3.5. Analysis of the Aptamer Folding Status Using a Single Aptamer Molecule Beacon

**Figure 7:**
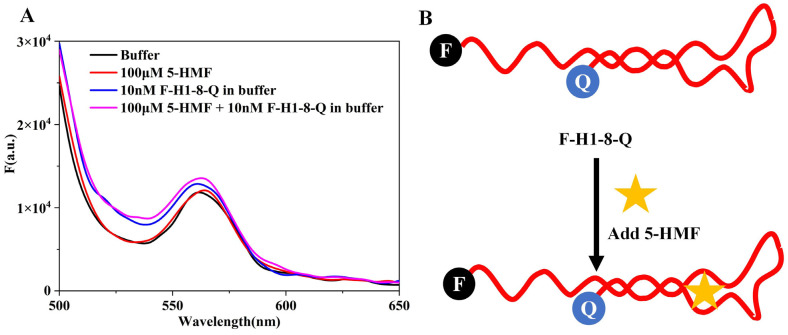
Analysis of F-H1-8-Q molecule beacon. (**A**) The spectra of F-H1-8-Q molecule beacon. (**B**) Principles of F-H1-8-Q molecule beacon.

In order to analyze the folding status of the aptamer, we designed a single aptamer molecule beacon (F-H1-8-Q), and the results are shown in Figure 7A. The maximum absorption wavelength of 6-FAM was 520 nm, but we could not detect the fluorescence peak at 520 nm for buffer and 10 nM F-H1-8-Q. This result indicated that the H1-8 aptamer was closed, and BHQ1 quenched the fluorescence of 6-FAM. When we added 5-HMF to the reaction system, the spectra of the (5-HMF+F-H1-8-Q) group were similar to the F-H1-8-Q group. It indicated that the H1-8 aptamer always closed whether 5-HMF was added or not. The 5-HMF did not have fluorescence signal, and it may not interfere in the application of F-H1-8-Q molecule beacon. According to these results, we deduced the detection principles of F-H1-8-Q, as shown in Figure 7B. Regardless of the 5-HMF adding or not, the F-H1-8-Q was closed, and we did not design this biosensor to detect 5-HMF in food.

### 3.6. Matrix Interference Analysis

**Figure 8:**
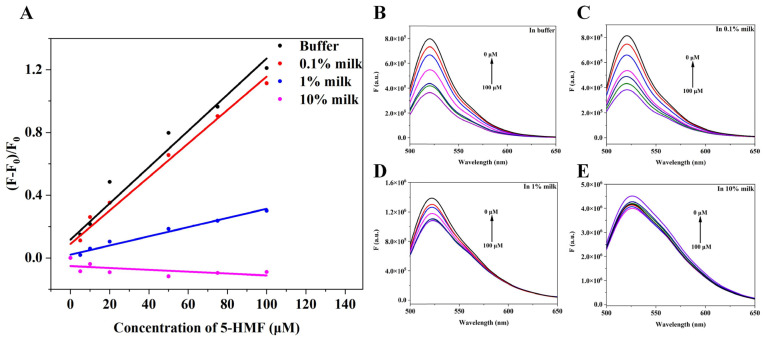
Matrix interference analysis. (**A**) The relationship between change of fluorescence intensity and concentration of 5-HMF in different matrix. (**B**) The fluorescence spectra of the quenching sensor in buffer. (**C**) The fluorescence spectra of the quenching sensor in 0.1% milk matrix. (**D**) The fluorescence spectra of quenching biosensor in 1% milk matrix. (**E**) The fluorescence spectra of the quenching sensor in 10% milk matrix.

The application of the quenching biosensor in this research was to detect 5-HMF in milk, and we studied the relationship between change of fluorescence intensity and concentration of 5-HMF. The results are shown in Figure 8. When in buffer, 0.1% milk, and 1% milk, the concentration of 5-HMF positively correlated with the change of fluorescence intensity (Figure 8A), but in 10% milk, the concentration of 5-HMF was not correlated with change of fluorescence, indicating that the high concentration of milk matrix interferes with the quenching biosensor. Therefore, we could detect the 5-HMF in 0–10% milk matrix.

## 4. Discussion

Aptamers are single-strand DNA or RNA functional nucleic acid molecules screened by SELEX. There are several reported SELEX technologies in the field of food safety, such as GO-SELEX, Beads-SELEX, and so on. Capture-SELEX is a widely used method to select aptamers specific for small molecules, because the lack of several active binding sites makes it difficult for screening aptamers specific to small molecules. In this capture-SELEX, we did not modify the target, and the ssDNA library was immobilized in solid phase carrier. The target induced the ssDNA to fall off when ssDNA bound with the target. Compared with the previous target immobilized SELEX, this method is simple, rapid and has a high success rate. After the 18th round selection, the high-throughput sequencing was used to sequence the enriched ssDNA library. The results of HTS were interesting, because there were four families with a homology rate of 90%. We selected five aptamers from the highly homologous families to identify their affinity. In the process of aptamer screening, the critical step was characterization of binding affinity, which could be assessed using various methods, such as surface plasmon resonance (SPR) [32], biomolecular layer interferometry (BLI) [33], ITC [34], electrophoretic mobility shift assay (EMSA) [35], microscale thermophoresis (MST) [36], capillary electrophoresis (CE) [37], and fluorescence measurement [38]. Among them, ITC is a classical method for analysis of molecular interaction between aptamers and small molecules [35]. In our study, ITC results showed that there were three candidate aptamers specific for 5-HMF with *K*_d_ of 2.46–12.40 μM. In order to verify the specificity, we further titrated FF to the three novel aptamers. We did not observe the binding heat, indicating the specific binding between 5-HMF and the three novel aptamers. Then, according to the structure difference between H1 and H3, we shortened the H1 aptamer to its four mutants (H1-8, H1-12, H1-14, H1-21). We discovered the relationship between secondary structure and binding activity of H1 and its mutants. This is a simple method for post-SELEX using the secondary structure to guide the aptamer truncation. All of the previous research did not select the aptamers specific for 5-HMF. It indicated that the aptamers specific for 5-HMF in this study were novel.

The mechanism of fluorescence quenching biosensor uses 6-FAM as the fluorescent probe and BHQ1 as the quenching probe through non-radiative coupling. It is a sensitive, reliable analytical technique and widely used in biological analysis [39]. The fluorescence quenching biosensors are a competitive alternative technology for rapid, ultrasensitive, reliable, and specific detection of small molecule residues [40]. Structural transition quenching biosensors of two DNA strands have been widely reported [27], where FAM fluorophore are labeled at the 5′ end of the aptamer, and a quenchers are labeled at the 3′ end of a 12-mer DNA. In the presence of small molecules, aptamer binding will result in the release of the quencher labeled strand and fluorescence enhancement. In our study, we also used this method to establish the fluorescence quenching biosensor. Since different ratios led to different fluorescence changes, we optimized the ratio to 2 (quencher):1(fluorophore), which saved the quencher DNA and ensured fluorescence intensity.

The fluorescence quenching technology enables quantitative analysis of molecular dynamics in biophysics and molecular biology [41]. We traced the dynamics of the fluorescence quenching biosensor response to observe whether 5-HMF could cause changes of fluorescence intensity after adding 5-HMF. We found that the changes of fluorescence were obvious when titration experiments with different concentration of 5-HMF were carried out. The fluorescence intensity was stable when 5-HMF reacted with the biosensor for 30 min. This result indicated that the reaction was equilibrium after 30 min incubation. Then, a different concentration of 5-HMF was titrated to the fluorescence quenching biosensor, and the emission spectra were collected. It was observed that the fluorescence intensity was enhanced after the addition of 5-HMF. We collected the fluorescence intensity at 520 nm and analyzed the relationship between changes of the fluorescence intensity and 5-HMF. We found the *K*_d_ value of about 64 μM, which is different from the *K*_d_ value using the ITC method. It may be the different principles between the fluorescence quenching biosensor and the ITC method, which is due to the different *K*_d_ value. In the standard, the residue limit of 5-HMF was 317 μM, which was higher than the maximum detected concentration of the fluorescence quenching biosensor, indicating that the fluorescence quenching biosensor is suitable for detection of 5-HMF in real samples.

The aptamer fold status is critical for biosensor designation, and we added the 6-FAM and BHQ1 labels at the 5′ and 3′ end of H1-8, respectively. The results showed that the fluorescence intensity was not changed whether 5-HMF was added or not, which indicated that the H1-8 aptamer was an end-closed aptamer. It was not suitable to design a single-strand biosensor because the fold status at the end of H1-8 aptamer did not change whether the target was added or not. Therefore, the double-strand fluorescence quenching biosensor is more universality than the single-strand fluorescence quenching biosensor, and the single-strand fluorescence quenching biosensor will be worked for special formation aptamer.

## 5. Conclusions

In conclusion, we selected three high-quality DNA aptamers specific for 5-HMF with excellent binding affinity and specificity. The selection results indicated the importance of ssDNA library enrichment for conveniently picking aptamers from the last round sequencing library. Comparing the H1 and H3 aptamers, there are five different bases in the third ring region, and cutting the fixed area improved the binding affinity, but breaking the differential base region would affect the binding affinity. The LOD of the fluorescence quenching biosensor for 5-HMF was 11.80 μM and could not bind analogue FF, allowing the detection of 5-HMF, specifically. At 0.1% and 1% milk matrix, the fluorescence quenching biosensor could accurately measure the 5-HMF concentrations. Given the importance of 5-HMF in food detection, we expect that these aptamers will be used as another important recognition element for developing biosensors.

## Figures and Tables

**Table 1:** Aptamers and its mutants sequences.

Name	Sequences (5′-3′)	Minimum Free Energy (kcal/mol)
H1	GACGACGTAGCGGCGGTGTGGGCATTTTGGGCTAAAGTCGTCCCGATGCT	−6.13
H2	GACGACTGTGGGCTGCCAAGGGCGCGTAGGGCGAAAGTCGTCCCGATGCT	−9.84
H3	GACGACTAAGCGGCGGTGTGGGCATTTTGGGCTTTTGTCGTCCCGATGCT	−6.56
H7	GACGACGTCCCGATGCTGCAATCGTAAACCCGTTATGTCGTCCCGATGCT	−4.15
H10	GACGACTAGAGGGGGCCCGTTATTCGGTGATGTCTTGTCGTCCCGATGCT	−7.52
H1-8	GACGACGTAGCGGCGGTGTGGGCATTTTGGGCTAAAGTCGTC	−5.9
H1-12	CGACGTAGCGGCGGTGTGGGCATTTTGGGCTAAAGTCG	−3.16
H1-14	GACGTAGCGGCGGTGTGGGCATTTTGGGCTAAAGTC	−0.99
H1-21	GTAGCGGCGGTGTGGGCATTTTGGGCTAA	−0.95

**Table 2:** Comparison of 5-HMF detection methods.

Detection Method	Detection Time (min)	Linear Range (μM)	LOD (μM)	Reference
Enzyme-linked immunosorbent assay	90	0.79–317	0.16	[28]
Ultra-high-performance liquid chromatography	-	9.52–634.92	1.19–3.97	[14]
High-performance liquid chromatography	70 (Including equilibrium time)	0.79–79.37	0.08–0.40	[29]
UV-Visible Spectrophotometric Method	-	7.94–158.73	-	[30]
High-performance liquid chromatography	48 (Including equilibrium time)	0.56–17.46	0.56	[31]
Biosensor	35	0–75	11.80	This study

## Data Availability

Data sharing is not applicable to this article.

## Supplementary Materials

The following supporting information can be downloaded at: https://www.mdpi.com/article/10.3390/bios13050564/s1, Figure S1: standard curve of Q-PCR; Table S1: DNA sequences from reference [42] used for aptamer selection; Table S2: selection conditions.
