# Supplementary material for: Selection of a Novel DNA Aptamer Specific for 5-Hydroxymethylfurfural Using Capture-SELEX
Source: Biosensors (Basel). 2023 May 22;13(5):564. doi: 10.3390/bios13050564 (PMC10216551; doi:10.3390/bios13050564)
Supplement: Supplementary file 1 [file biosensors-13-00564-s001.zip › biosensors-2375276-supplementary.pdf]

Supporting Information

Table S1. DNA sequences from reference [1] used for aptamer selection.

Name	Sequences (5′-3′)
N_{30}Library	GGAGGCTCTCGGGACGAC$(N)_{30}$GTCGTCCCGATGCTGCAATCGTAA
F(Lib30)	GGAGGCTCTCGGGACGAC
R(Lib30)	TTACGATTGCAGCATCGGGACG
FAM-F(Lib30)	FAM-GGAGGCTCTCGGGACGAC
Biotin-R(Lib30)	Biotin-TTACGATTGCAGCATCGGGACG
(lib30)CS-Biotin	5'-GTCGTCCCGAGAGCCATA-BioTEG-3'

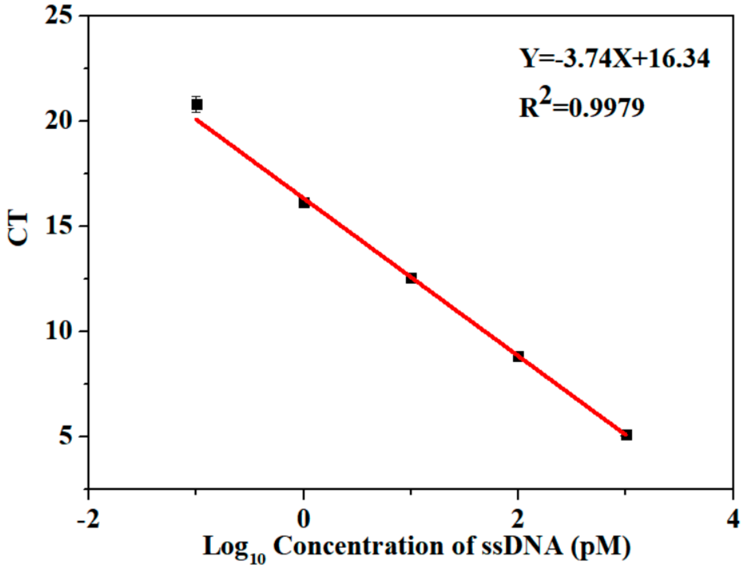

Figure S1. Standard curve of Q-PCR.

Table S2. Selection conditions.

Round	N30 Library (pmol)	5-HMF (mM)	FF (mM)
1	500	100	
2	400	100	
3	300	100	
4	100	100	
5	100	10	
6	100	10	
7	100	10	
8	100	10	
9	100	10	
10	100	1	
11	100	1	
12	100	1	
13	100	1	
14-	100		1
14+	100	1	
15-	100		1
15+	100	1	
16-	100		1
16+	100	1	
17-	100		10
17+	100	1	
18-	100		10
18+	100	1	

List of the library and target concentration used for each round of selection. – means negative selection and + means positive selection.

References

1. Nakatsuka, N.; Yang, K.A.; Abendroth, J.M.; Cheung, K.M.; Xu, X.B.; Yang, H.Y.; Zhao, C.Z.; Zhu, B.W.; Rim, Y.S.; Yang, Y.; et al. Aptamer-field-effect transistors overcome Debye length limitations for small-molecule sensing. *Science* **2018**, *362*, 319–325.
